# Space Technologies for Sustainable Heritage: 10th anniversary of HIST

**DOI:** 10.1186/s40494-021-00630-0

**Published:** 2021-12-07

**Authors:** Fulong Chen, Xinyuan Wang, Jie Liu, Shaobo Liu

**Affiliations:** International Centre on Space Technologies for Natural and Cultural Heritage under the Auspices of UNESCO, Beijing, 100094 China

World Heritage sites, Biosphere reserves and UNESCO Global Geoparks are three primary categories of UNESCO-designated sites. As irreplaceable sources of life and inspiration, heritage is our legacy from the past, what we live with today, and what we pass on to future generations [1]. Strengthening efforts to protect and safeguard world heritage sites has been among the targets of the United Nations (UN) Sustainable Development Goal (SDG) 11 in recognition of heritage’s role in contribution to a more resilient society and more sustainable liveability in cities. However, due to a combination of natural processes, especially the increasing frequency of disasters (i.e., flood and earthquake) and anthropogenic activities, including aggravated human disturbances (urbanization and resource exploitation), sustainable development of heritage properties is challenging. Therefore, there is a growing need to utilize emerging sciences and technologies to resolve the contradictions between conservation and utilization of these valuable cultural and natural resources.

Taking into consideration the interdisciplinary nature of heritage studies, scientists, engineers, managers and relevant stakeholders with diverse knowledge advocate a more cooperative approach to develop comprehensive, yet practical, solutions and applications. Space technologies, including remote sensing, Geographic Information System (GIS), Global Navigation Satellite System (GNSS), along with big data, cloud computation, artificial intelligence, 5G communications etc., provide a new paradigm of scientific and technological innovation, with the potential to transform the way of social production and people’s life.

The relevance and performance of space technologies in managing and monitoring heritage sites has already been established. Applications for space technologies in heritage conservation enable innovative solutions primarily in three aspects. First, the core elements of heritage ontology along with surrounding environments can be digitalized to develop seamless virtual representations that help to archive valuable archaeological assets, improve resource management, enable digital forensic studies and data intensive investigations utilizing modern analytical techniques and methods. Secondly, space-air-ground stereoscopic observation enables high resolution monitoring of heritage sites to sense minute changes and perceive historical and ongoing transformations in near real-time providing comprehensive information to develop practical solutions. Third, multisource spatiotemporal data and models can be collected, and integrated into a smart management system.

Additionally integrating big data analytics, along with simulation/prediction, virtual reality/augment reality, socio-economic and geo-science process model, enhances the capacity to optimize management countermeasures to ensure sustainable development of these heritage sites. These technological resources also help to promote the Outstanding Universal Value (OUV) of heritage sites through digital communication platforms improving knowledge, education, awareness, and recognition of the OUV, and improving community involvement sustainable conservation of UNESCO-designated sites, ensuring a transformation towards a resilient society.

The International Centre on Space Technologies for Natural and Cultural Heritage (HIST) under the auspices of UNESCO was proposed to UNESCO by the Chinese Academy of Sciences (CAS) in May 2007. The proposal was approved by the 35th General Conference of UNESCO in October 2009, and ratified by China’s State Council in April 2011. On 24 July, 2011, the launching ceremony of HIST was held in Beijing. HIST is devoted to developing and utilizing space technologies for the identification, conservation, monitoring and management of UNESCO-designated sites to support UNESCO and its Member States in the implementation of the World Heritage Convention and providing support for the implementation of the UN 2030 Agenda for delivering Sustainable Development Goals (SDGs).

Since its inauguration in July 2011, HIST has played a vital role in assisting UNESCO and its Member States in the conservation of UNESCO-designated sites. It has conducted 70 international conservation projects such as Remote Sensing for Environment of Angkor Site, Monitoring Landscape Evolution and Abnormal Deformations of the pagodas in Bagan World Heritage site, Monitoring and Analysis of Changes in the Forest Cover Loss in Asian Elephants’ Habitats in Sri Lanka, Digital Characterization and Preventive Conservation of the Ming Great Wall with 20 UNESCO Member States, like Cambodia, Tunisia, Italy, Brazil, and strengthened capacity building through cultivating 20 doctoral students from developing countries and training 300 heritage site managers, technicians, researchers and decision-makers from UNESCO Member States. Meanwhile, HIST has pioneered the building of a new discipline-Space Archaeology. One of the landmark achievements is the publication of the *Introduction to Space Archaeology (Chinese Version),* which is the first book of its kind that reviewed space information technology applications for archaeology and introduced different space information techniques used for archaeology application and discussed the potential to establish a new discipline: Space Archaeology. Another prominent case is that HIST worked with other international scientists to make the discovery of 10 new archaeological sites in Tunisia dating to ancient Rome using Chinese space technology, which won the plaudits from Tunisian Ministry of Culture and Chinese Academy of Sciences. In addition, HIST has also expanded its global network with more than 30 UNESCO Member States and international organizations such as IUCN, ICOMOS, ICCROM. In the past decade, HIST has made outstanding achievements, received international recognition and wide praise from UNESCO and other international organizations as well as relevant governments and institutions across the globe.

Supported by the Bureau of International Cooperation, Chinese Academy of Sciences, HIST has joined hands with *Heritage Science*, part of Springer Nature, to launch the special issue (article collection) to commemorate its 10th anniversary. This special issue presents the latest academic achievements from HIST researchers and its international partners. It groups a selection of eleven papers to illustrate the uses of space technology in the characterization, monitoring and assessment of heritage sites. These research studies not only highlight the necessity of this technology by emphasizing its benefits in first-hand data collection at macro–micro scales, but also reveals its advantages in sustainable conservation of heritage properties by means of spatiotemporal analysis and scientific findings.

As the one of the four ancient civilizations worldwide, the Chinese civilization is quite unique and one that has lasted continuously for thousands of years. Cultural heritage sites are witnesses to our history, a mirror to reflect upon our past. Yan et al. [2] utilized GIS spatial analysis to establish a spatiotemporal database of ancient cities in the late Yangshao, Longshan, as well as Xia and Shang Dynasties in the Central Plains (one of the cradles of the Chinese civilization) to figure out the relationship between the ancient city distribution, geographical environment and the evolution of ancient city's shapes and sizes. Traditional villages are the living carrier for the Chinese culture demonstration and inheritance. These properties are facing sustainable development challenges due to the urbanization and the global change. Zhang et al. [3] studied silo-caves that are a unique human habitation form on the Loess Plateau in northern China (dating back to 7000 years) and the “living fossils for the history of dwellings”. The historical evolution of this property analysed by remote sensing and GIS techniques provided us sustainable routines from perspectives of administrative policy and planning, people’s awareness of cultural heritage and etc. On the other hand, the universal value of traditional villages and settlements was presented in Hu et al. using the proposed “Cultural Landscape Genes of Traditional Settlements” in theoretically using the digitalized symbolization methods [4]. Li et al. presents a case study of Gulangyu Island historical international community, the spatial evolution path and the multicultural integration of local communities was demonstrated and interpreted [5] based on the interpretation of historical maps and the integration of historical geographic information. Satellite sensors vividly observes the uniform geological and geomorphological landform of the Earth synoptically using multi-spectral remote sensing images. Fu et al., utilized the aforementioned capability to characterize the geological significance of UNESCO Global Geoparks in terms of their scientific quality, rarity, aesthetic appeal and the value for tourism [6].

On site or space-based monitoring and surveillance is the most effective way to evaluate the condition of heritage sites as well as their surrounding environment. For those inaccessible locations (i.e., the lockdown due to COVID-19 and complex landscapes), the prominent role of remote sensing for heritage monitoring is essentially important and irreplaceable. Owing to the technological advance of active and passive Earth observation sensors along with intelligent processing chains (i.e., random forest classifier) and the emerging of open-source data (i.e., European Copernicus Sentinel-1 and Sentinel-2), remote sensing has been applied as a systematic observation tool for stakeholders to map drastic or slowly driven landscape changes towards the better management of these sites and their surroundings [7]. In case the urgent monitoring and assessment task is required from UNESCO, the status of large-scale World Heritages can be quantitatively characterized using the thematic maps obtained from time series analysis of remote sensing images, such as the assessment conducted for the endangered UNESCO World Heritage site of East Rennell, the Solomon Islands [8]. More recently, natural heritage sites and biosphere reserves are recognized as sensitive areas for climate change and ecological degradation. In Li et al. [9], the dynamic response of the vegetation carbon storage to the land use/cover was assessed by synergistically using remote sensing images and temperature and precipitation data in the Sanjiang Plain in China. Apart from the visible environmental changes, the imperceptible deformations for the decline of architectural heritages were monitored in Xu et al. using multi-temporal synthetic aperture interferometry (MTInSAR) approaches taking advantage of the capability in estimating spatiotemporal deformations with accuracy up to millimeters, as verified by the comparative case study of the Shanhaiguan section of the Great Wall (China) [10].

The paradigm of scientific discovery has transformed from statistical modelling to the data-driven and knowledge mining in the era of big data. Considering the transboundary and interdisciplinary characteristics of heritage studies, the potential of big data science in the sustainable conservation and smart management of heritage sites needs to be fully exploited. In Liang et al. [11], the spatiotemporal evolution pattern of world cultural heritage (869 inscriptions up to 2019) was depicted using the GIS-based tool. The result reveals that the implementation of heritage policy impacts the inscription number of heritage sites distributed in diverse economic development regions, implying the necessity for the strategy optimization to fully guarantee the category and regional-distributed balance of inscribed heritage sites in order to achieve the sustainable development goals globally.

In general, the digital technology and data aggregation are the foundation for the data-driven knowledge mining. In the smart management of Mount Lushan project, Cai et al. evaluated the performance of digital technologies including oblique aerial photography, 3D laser scanning and 360-degree panorama imaging and synergistically utilized them to integrate all elements (including social-economic data) into a virtual tourism subsystem. This pilot application is a representative demo to overcome the shortcoming of heritage protection and heritage tourism marketing [12].

Looking forward, future researches on the application of space technologies to heritage conservation will be conducted in six major fields: (a) heritage-related multi-source data access, big data processing and analytic techniques, (b) fine-precise heritage conservation methods; (c) digital heritage technological system based on high and newly emerging technologies; (d) blending and presentation techniques for heritage utilization; (e) the formulation of different standards for heritage property conservation and digital conservation; (f) participatory heritage education, social governance pathways and methods.

In the next decade, HIST would like to strengthen partnerships with UNESCO and its Member States and advisory bodies as well as other international organizations to meet strategic demands for the conservation and sustainable development of UNESCO-designated sites. It calls for global stakeholders to make concerted efforts to integrate science, technology and engineering to develop a system of theoretic framework and technological methodology, to further build space archaeology paradigm, to construct an international digital world heritage platform for scientific research and technological application by integrating database, monitoring, evaluation, presentation and decision support, and to establish a whole chain of digital heritage with a mix of data access, processing, analysis, presentation, application and industrialization, thus helping UNESCO and its Member States to achieve heritage-related SDGs.
